# Low Valine Serum Levels Predict Increased 1-Year Mortality in Acute Heart Failure Patients

**DOI:** 10.3390/biom13091323

**Published:** 2023-08-29

**Authors:** Iva Klobučar, Luka Vidović, Ilona Arih, Margarete Lechleitner, Gudrun Pregartner, Andrea Berghold, Hansjörg Habisch, Tobias Madl, Saša Frank, Vesna Degoricija

**Affiliations:** 1 Department of Cardiology, Sisters of Charity University Hospital Centre, 10000 Zagreb, Croatia; iva.klobucar@gmail.com; 2 Department of Medicine, Sisters of Charity University Hospital Centre, 10000 Zagreb, Croatia; lukavidovic70@gmail.com (L.V.); vdegoric@mef.hr (V.D.); 3 School of Medicine, University of Zagreb, 10000 Zagreb, Croatia; ilona.arih@gmail.com; 4 Gottfried Schatz Research Center, Molecular Biology and Biochemistry, Medical University of Graz, 8010 Graz, Austria; margarete.lechleitner@medunigraz.at (M.L.); hansjoerg.habisch@medunigraz.at (H.H.); tobias.madl@medunigraz.at (T.M.); 5 Institute for Medical Informatics, Statistics and Documentation, Medical University of Graz, 8036 Graz, Austria; gudrun.pregartner@medunigraz.at (G.P.); andrea.berghold@medunigraz.at (A.B.); 6 BioTechMed-Graz, 8010 Graz, Austria

**Keywords:** acute heart failure, valine, mortality, NMR spectroscopy, prognostic biomarkers, risk, metabolomics

## Abstract

Considering the relationship between disease severity and the extent of metabolic derangement in heart failure, we hypothesized that the serum levels of metabolites may have prognostic value for 1-year mortality in acute heart failure (AHF). The AHF study was a prospective, observational study enrolling consecutive patients hospitalized due to AHF. Metabolites were measured in serum collected at admission using NMR spectroscopy. Out of 315 AHF patients, 118 (37.5%) died within 1 year after hospitalization for AHF. The serum levels of 8 out of 49 identified metabolites were significantly different between patients who were alive and those who died within 1 year after hospitalization for AHF. Of these, only valine was significantly associated with 1-year mortality (hazard ratio 0.73 per 1 standard deviation increase, 95% confidence interval: 0.59–0.90, *p* = 0.003) in the multivariable Cox regression analyses. Kaplan–Maier analysis showed significantly higher survival rates in AHF patients with valine levels above the median (>279.2 µmol/L) compared to those with valine levels ≤ 279.2 µmol/L. In a receiver operating characteristics curve analysis, valine was able to discriminate between the two groups with an area under the curve of 0.65 (95% CI 0.59–0.72). We conclude that valine serum levels might be of prognostic value in AHF.

## 1. Introduction

Heart failure (HF) is the final stage of various cardiovascular diseases. As a complex clinical syndrome, HF affects not only the heart but also other organs, in particular the liver, kidney, and intestine. Despite improvements in the treatment of cardiovascular diseases and the overall clinical management of HF patients, the incidence of HF has been increasing and is still associated with poor patient outcomes [1]. 

Left ventricular dysfunction and a consequent tissue hypoperfusion, ischemia, and hypoxia, as well as congestion as a consequence of right-sided HF, are the principal triggers of metabolic dysfunction, an inherent feature of the HF pathophysiology [2]. The diminished motility of the hypoperfused and congested gut alters the composition of the gut microbiota and promotes local inflammation, leading to decreased nutrient absorption as well as increased gut mucosa permeability [3,4]. The latter promotes translocation of the gut microbiota and their toxins into systemic circulation, leading to low-grade persistent systemic inflammation and the promotion of insulin resistance, a principal metabolic feature of the HF pathophysiology [4]. Both systemic inflammation and insulin resistance, together with natriuretic peptides and catecholamines, whose levels are increased due to heart wall distension and renal hypoperfusion, promote lipolysis, proteolysis, and oxidative stress, the hallmarks of metabolic dysfunction in HF [5]. Metabolic dysfunction and catabolic dominance in HF are further intensified by a reduced appetite, the diminished biosynthetic capacity of the hypoperfused and/or congested liver, altered gut microbiota metabolites, and a progressive worsening of renal function [2].

Acute heart failure (AHF) is characterized by either the rapid onset of or worsening of the signs and symptoms of HF [6]. Despite continuous efforts to improve multivariable predictive models comprising serum biomarkers and patients’ clinical characteristics, the estimation of risk is difficult, and the mortality rate in AHF is still unacceptably high [7]. Therefore, identification and implementation of new serum biomarkers reflective of the underlying AHF pathophysiology may help improve the estimation of risk and thus the outcome of AHF patients.

Considering that the extent of the metabolic perturbation is closely related to the hemodynamic impairment, it is conceivable that the metabolomic profile of the patients’ serum at hospital admission resembles the severity of AHF and may therefore have a prognostic capacity in AHF. The aim of the present study was therefore to employ nuclear magnetic resonance (NMR) spectroscopy to identify serum metabolites that are prognostic for 1-year mortality in AHF patients. 

## 2. Methods

### 2.1. Study Design and Patients

The AHF study was a prospective, observational study enrolling patients who presented to the emergency department and required hospitalization due to clinical signs and symptoms of AHF. The diagnosis of AHF and the treatment of all patients were done according to the valid definition and guidelines given by the European Society of Cardiology at the time of study implementation [6]. Inclusion and exclusion criteria, as well as the study flow chart (Supplementary Scheme S1), have been described in our previous reports [8,9]. Participants were followed up for one year after the index AHF hospitalization, and the primary endpoint was all-cause mortality. 

Written informed consent was obtained from each enrolled patient prior to any study procedure. The study was conducted in adherence to the Good Clinical Practice guidelines and the principles of the Declaration of Helsinki [10]. The study was approved by the local ethics committees of the Sisters of Charity University Hospital Center, Zagreb, Croatia (EP 2258/18-10), and the Medical University of Graz, Austria (EK 33-258 ex 20/21).

### 2.2. Laboratory Procedures

Blood samples from each participant were collected at the time of admission to the hospital. The procedure for blood sample collection and the standard laboratory methods have been described in our previous reports [8,9]. 

### 2.3. Metabolites Profiling by Nuclear Magnetic Resonance (NMR) Spectroscopy 

Blood serum and low-molecular-weight metabolites were analyzed by NMR spectroscopy. The analytes (in total, 49) comprise amino acids and their derivatives, carboxylic acids, ketone bodies, and monosaccharides. The preparation of samples was done as previously described [11]. Briefly, serum samples were thawed, 200 µL of each sample were mixed with 400 µL of ice-cold methanol in order to precipitate and inactivate proteins, and they were stored at −20 °C for at least 30 min. Following centrifugation at 10,000× *g* at 4 °C for 30 min, supernatants were lyophilized and resuspended in 500 µL of NMR buffer (80 mM sodium phosphate (Carl Roth, Karlsruhe, Germany), pH 7.4, deuterium dioxide (Eurisotop, St-Aubin Cedex, France), 4.6 mm deuterated trimethylsilylpropionic acid (TSP; Eurisotop), transferred into 5 mm glass tubes, and placed into a SampleJet rack (both from Bruker, Rheinstetten, Germany). Proton spectra were obtained at a constant temperature of 310 K on an AVANCE™ Neo Bruker Ultrashield 600 MHz spectrometer equipped with a TXI probe head using a Carr–Purcell Meiboom–Gill (CPMG) pulse sequence with presaturation during the relaxation delay (Bruker: cpmgpr1d) to achieve water suppression. Sample spectra were further processed using Matlab^®^ vR2014a (Mathworks, Natick, MA, USA) scripts. Briefly, NMR data were imported into Matlab^®^, regions around water, TSP, and remaining methanol signals were excluded, and probabilistic quotient normalization was performed to correct for sample metabolite dilution. Evaluated peak areas were normalized to concentrations of metabolite concentration standards. 

### 2.4. Statistics

Metric parameters were summarized as means and standard deviations (SD) or medians and interquartile ranges (IQR), whereas absolute and relative frequencies were used to describe categorical parameters. Differences between patients who were alive and those who died within 1 year, as well as between groups defined by various clinical characteristics, were tested with the *t*-test, Mann–Whitney U test, or Fisher’s exact test. Univariable and multivariable Cox regression analyses were used to examine the prognostic value of the metabolites for 1-year mortality. The multivariable analyses were adjusted for age, sex, body mass index (BMI), mean arterial pressure (MAP), estimated glomerular filtration rate (eGFR), blood urea nitrogen (BUN), C-reactive protein (CRP), N-terminal pro-brain natriuretic peptide (NT-proBNP), hemoglobin, alanine aminotransferase (ALT), albumin, and total cholesterol. Sensitivity analyses were performed by replacing total cholesterol with LDL cholesterol, signs of venous volume overload, and the previous use of ß-blockers or angiotensin-converting enzyme inhibitors, as well as by omitting or replacing albumin with signs of venous volume overload in the multivariable Cox regression analyses. Before the survival analyses, the metabolite concentrations were log-transformed to overcome the skewness of the metabolite distributions. Results are presented as hazard ratios (HR) and the respective 95% confidence intervals (CI) per 1-SD increase. We performed Kaplan-Meier survival analysis for valine concentrations above vs. below the median and compared them using the log-rank test. A receiver operating characteristic (ROC) curve analysis was performed to assess the prognostic ability of valine. The Spearman correlation coefficient was used to assess correlations between valine and various clinical and laboratory parameters; the results are presented in a heatmap. A *p*-value < 0.05 was generally considered significant. However, in the initial analyses (Mann-Whitney U test) identifying metabolites whose levels were significantly different between patients who were alive and those who died within 1 year, a Bonferroni correction was applied to correct for multiple testing, and thus a *p*-value < 0.001 (0.05/49) was considered significant. R version 4.1.0 and MetaboAnalyst 5.0 were used for these analyses. We did not impute any missing values. Our data allow for the detection of significant group differences between patients alive vs. deceased after 12 months (*n* = 197 vs. *n* = 118) for effect sizes of 0.38 or for correlation coefficients within the whole cohort (*n* = 315) of at least 0.18, with a power of 90%.

## 3. Results

### 3.1. Clinical Characteristics, Chronic Medication, and Standard Laboratory Parameters

A total of 315 patients hospitalized due to AHF were enrolled in the study. The mean (±SD) age was 74.2 ± 10.5 years, and 136 (43.2%) were female. Within 1 year after the index AHF hospitalization, 118 (37.5%) participants died. Differences in baseline clinical characteristics, chronic medication, and standard laboratory parameters at the time of hospital admission between these patients and those that survived have been described in our previous reports [8,9] and are shown in Table 1 and Table S1. 

### 3.2. Association between Metabolites and 1-Year Mortality in AHF Patients

**Figure 1:**
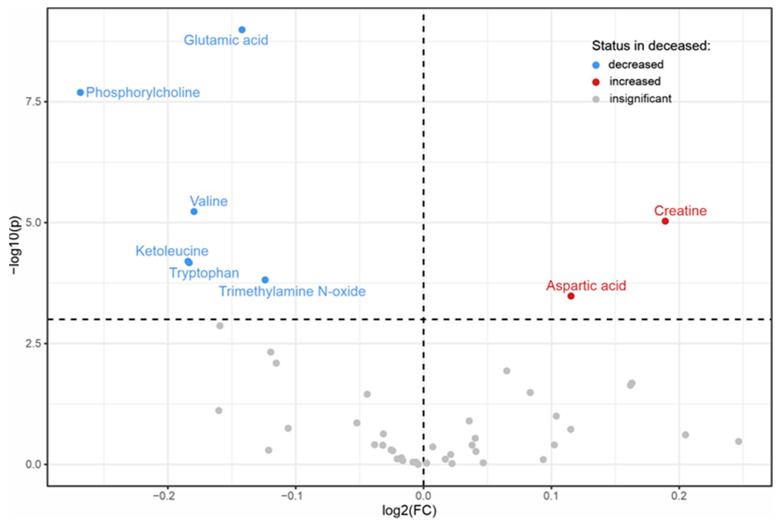
Volcano plot showing metabolites whose serum levels are significantly decreased, increased or not significantly different in patients who died compared to those who were alive within 1 year after hospitalization for AHF. Differences between the groups were tested with the Mann-Whitney U test. A *p*-value of <0.001 was considered significant after a Bonferroni correction for multiple testing.

After a Bonferroni correction for multiple testing, serum levels of 8 metabolites (out of 49 identified metabolites) were significantly different between patients who were alive and those who died within 1 year after hospitalization for AHF (Figure 1, Tables S2 and S3).

**Figure 2:**
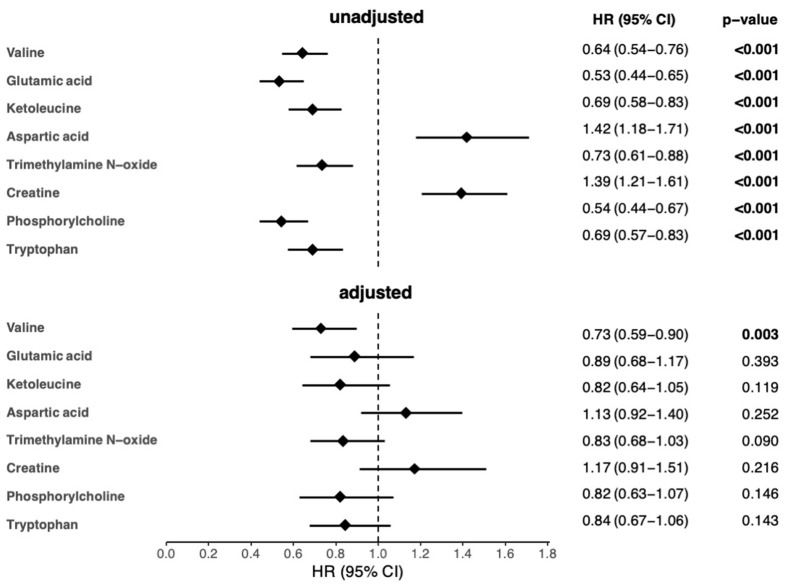
Cox regression analyses of metabolites as predictors of 1-year mortality in AHF patients. Metabolites (µmol/L) were log-transformed before Cox regression analysis. Results are presented as hazard ratios (HR) and the respective 95% confidence intervals (CI) per 1-SD increase. *p*-values < 0.05 were considered significant and are depicted in bold. In the adjusted model, age, sex, BMI, MAP, eGFR, BUN, CRP, NT-proBNP, hemoglobin, ALT, albumin, and total cholesterol were used as covariates. The total numbers of patients and events were 315 and 118, respectively, in the univariable analyses and 303 and 112, respectively, in the adjusted analyses. ALT, alanine aminotransferase; BMI, body mass index; BUN, blood urea nitrogen; C, cholesterol; CRP, C-reactive protein; eGFR, estimated glomerular filtration rate; HR, hazard ratio; MAP, mean arterial pressure; N, number of observations; NT-proBNP, N-terminal pro-brain natriuretic peptide; SD, standard deviation.

Accordingly, the 8 metabolites were significantly associated with 1-year mortality in the univariable Cox regression analyses (Figure 2). However, only the association of valine remained significant after adjustment for age, sex, and other clinical and laboratory parameters significantly associated with 1-year mortality in the univariable analyses (Figure 2 and Table S4). 

**Figure 3:**
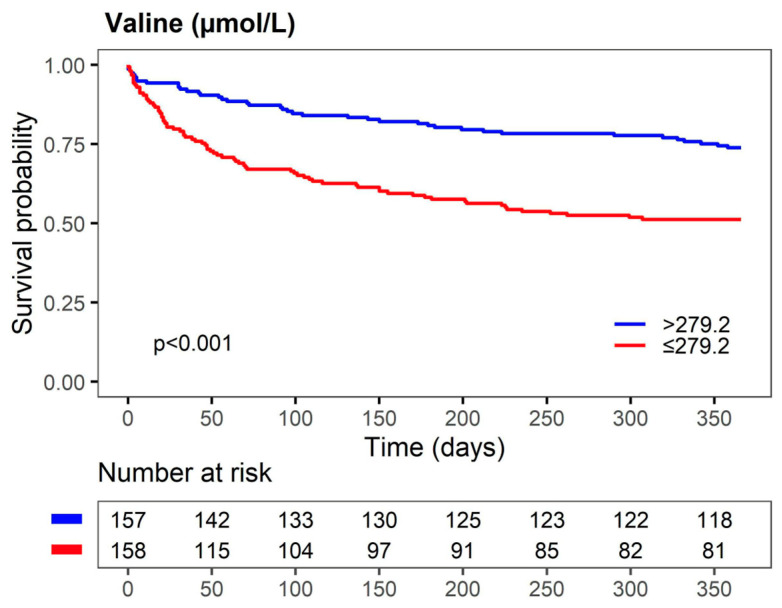
Kaplan-Meier survival curves for valine predicting 1-year mortality of AHF patients. The number of patients at risk at each time point is presented below the graph.

The replacement of total cholesterol with LDL cholesterol, signs of venous volume overload, ß-blockers, or angiotensin-converting enzyme inhibitors, as well as omitting or replacing albumin with signs of venous volume overload, had no effect on the association of valine with 1-year mortality (Table S5). Kaplan-Maier survival analysis to compare groups defined by the median valine level (279.2 µmol/L) revealed a significantly higher survival in AHF patients with valine serum levels > 279.2 µmol/L compared to those with ≤279.2 µmol/L (Figure 3). 

**Figure 4:**
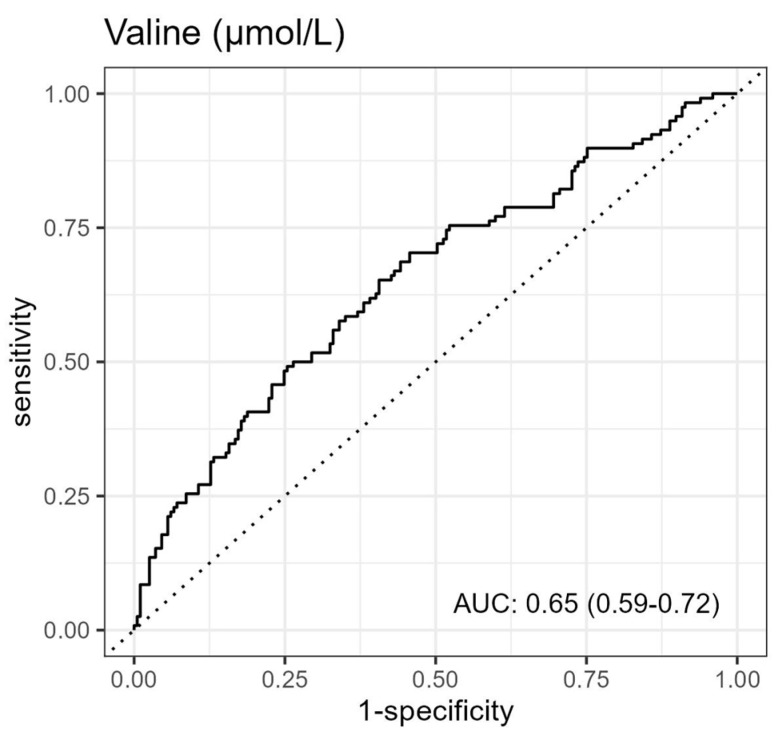
Receiver operating characteristics curve for prediction of death within 1 year of valine.

ROC curve analysis to assess the accuracy of valine to predict death within 1 year after hospitalization revealed an area under the curve (AUC) of 0.65 with a 95% CI of 0.59–0.72 (Figure 4). The accuracy of a panel of metabolites comprising valine and the other metabolites significantly associated with mortality in the univariable Cox regression analysis (Figure 2) revealed an AUC of 0.77 with a 95% CI of 0.72–0.82 (Figure S1). 

### 3.3. Correlation Analyses of Valine with Clinical and Laboratory Parameters

**Figure 5:**
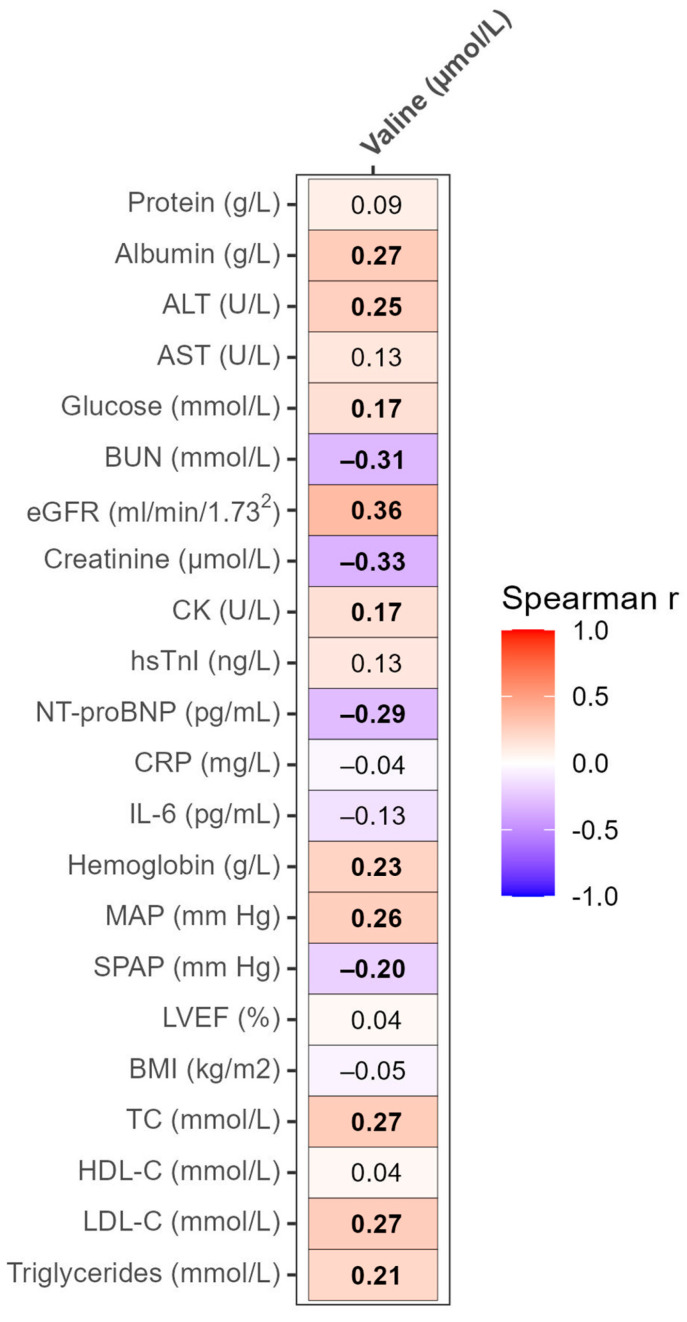
Heatmap of correlations of valine serum levels with laboratory and clinical parameters. Values presented are the Spearman correlation coefficients. *p*-values < 0.05 were considered significant and are depicted in bold. SPAP was measured in 259 patients; other analyses were based on 315 samples. ALT, alanine aminotransferase; AST, aspartate aminotransferase; BUN, blood urea nitrogen; CK, creatine kinase; CRP, C-reactive protein; eGFR, estimated glomerular filtration rate; HDL-C, high-density lipoprotein cholesterol; hsTnI, high-sensitivity troponin I; IL-6, interleukine-6; LDH, lactate dehydrogenase; LDL-C, low-density lipoprotein cholesterol; LVEF, left ventricular ejection fraction; MAP, mean arterial pressure; NT-proBNP, N-terminal pro-brain natriuretic peptide; SPAP, systolic pulmonary artery pressure; TC, total cholesterol.

Valine serum levels were significantly positively correlated with serum albumin, ALT, and glucose, as well as eGFR, creatine kinase (CK), hemoglobin, and MAP. Additionally, valine was significantly positively correlated with serum lipids and lipoproteins, including total cholesterol, low-density lipoprotein cholesterol (LDL-C), and triglycerides. Significant negative correlations were found between valine and BUN, creatinine, NT-proBNP, and systolic pulmonary artery pressure (SPAP) (Figure 5). 

### 3.4. Differences in Valine Serum Levels in Various Groups of AHF Patients

Valine serum levels were significantly higher in AHF patients with type 2 diabetes mellitus (T2D) and coronary artery disease (CAD), as well as significantly lower in patients with chronic kidney disease (CKD) and atrial fibrillation (AF) compared to AHF patients without these comorbidities (Table 2). Significantly lower valine serum levels were also found in patients with one or more signs of venous volume overload (peripheral edema, enlarged liver, ascites, or jugular venous distension) compared to those without. Similar valine serum levels were found in AHF patients with and without metabolic syndrome (MetS), as well as in those who developed AHF as a worsening of CHF compared with those with new-onset AHF (Table 2). 

## 4. Discussion

Here, we show for the first time the profound prognostic capacity of valine serum levels for 1-year mortality in AHF patients. Of note, 94% of our patients belonged to NYHA class IV. These results and a previously reported association of low valine serum levels with adverse events (all-cause mortality, re-hospitalization for worsening HF, and atrial fibrillation) in CHF patients who primarily belonged to NYHA classes II [12] or III and IV [13] may suggest the applicability of valine as a prognostic biomarker across the severity stages and types of HF. However, in an AHF cohort in which none of the patients belonged to NYHA class IV, ratios of leucine to phenylalanine and of branched-chain amino acids to aromatic amino acids, as well as plasma levels of leucine but not valine, were associated with cardiac death or hospitalization for worsening HF [14,15]. In the present study, associations of other metabolites with 1-year mortality did not remain significant upon adjustment for confounders significantly associated with 1-year mortality. This implies that, in contrast to the other tested metabolites, valine independently depicts various aspects of the underlying AHF pathophysiology as well as specifically reflects the severity of the disease and the detrimental impact of the failing heart on other organs and tissues in AHF patients. 

In the present study, valine was negatively correlated with NT-proBNP, a marker of HF and left ventricular dysfunction [16], as well as with BUN, one of the strongest predictors of adverse outcome in HF [17], and SPAP, which is increased secondary to the left ventricular dysfunction in HF [18]. This, together with positive correlations of valine with MAP, eGFR, and hemoglobin, which are negatively affected by the HF pathophysiology, highlights a negative relationship between valine serum levels and the severity of AHF. In contrast to the disease severity, there was no association of the indicators of disease chronicity with valine, as exemplified by the similar valine serum levels in our patients with new-onset AHF and those who developed AHF on top of CHF. In other words, it seems that pathophysiological mechanisms operative during the acute phase of HF are more potent modulators of valine serum levels than those operative during the chronic, but stable, phase of HF.

Reduced appetite and impaired intestinal absorption of nutrients due to congestion-induced edema, both resulting in poor nutritional states, are likely causes for the lower valine levels observed in AHF patients who died. Additionally, a reduction in valine-producing gut microbiota secondary to a global alteration in the gut microbiome composition, a consequence of reduced motility of the hypoperfused, inflamed, and edematous gut, might, at least in part, be responsible for the decreased valine supply to the systemic circulation [3,4]. Indeed, valine serum levels were lower in our AHF patients with signs of venous volume overload and were positively correlated with serum albumin and lipid levels, which are known to be negatively affected by a poor nutritional state, impaired intestinal absorption, and reduced biosynthetic capacity of the liver [19]. Moreover, decreased valine serum levels may reflect an overconsumption of branched-chain amino acids (BCAA) by the failing heart and the congested lungs, which increasingly use valine and other BCAA as building blocks for the tissue remodeling processes induced by altered hemodynamics [20]. 

Decreased CK levels indicate catabolic dominance and muscle wasting, the typical signs of advanced HF [21]. We observed a positive correlation between valine serum levels and CK, which was significantly lower in AHF patients who died compared to those who were alive 1 year after hospitalization for AHF. Since protein degradation and BCAA release due to a hypercatabolic state contribute to BCAA serum levels in HF patients [22], the lower valine serum levels in our patients who died within 1 year after hospitalization for AHF might also be ascribed to the diminished valine release from the wasted muscles. 

Cellular catabolism of BCAA includes deamination to yield branched-chain alpha-keto acids (BCKA), which undergo conversion mediated by BCKA dehydrogenase to the precursors of energy metabolism molecules such as acetyl-CoA and succinyl-CoA. While BCKA dehydrogenase kinase phosphorylates BCKA dehydrogenase and inactivates the enzyme, dephosphorylation by a specific phosphatase restores the enzyme activity [23,24]. Endotoxins and inflammatory cytokines, whose levels are increased in HF, are strong inducers of BCKA dehydrogenase activity in the skeletal muscle [23,25,26]. However, in the present study, valine serum levels were neither significantly correlated with IL-6 nor CRP, arguing against an increased valine breakdown by the skeletal muscle as a cause for the decreased valine serum levels in patients who died within 1 year after hospitalization for AHF. 

In line with previous studies, valine serum levels in the present study were higher in AHF patients with T2D compared to those without [27,28]. A positive correlation between valine and glucose observed in the present study most likely reflects regulation of these metabolites by the nutritional state and the efficacy of intestinal absorption. Additionally, considering the established association between BCAA and insulin resistance [23], the valine-induced impairment of insulin sensitivity, accompanied by an increase in glucose levels, might be a driver of the positive correlation between valine and glucose in our patients. Consistent with the negative impact of renal dysfunction on the serum levels of BCAA and branched-chain α-ketoacids (BCKA) [29], valine levels were lower in AHF patients with CKD and positively correlated with eGFR. Higher valine serum levels in our AHF patients with CAD compared to those without are in agreement with the positive association of valine and other BCAAs with CAD described in a previous study [30]. 

In experimental mouse models of HF induced either by pressure overload or myocardial infarction, cardiac BCAA catabolism was impaired and accompanied by the cardiac accumulation of BCAA and BCKA, increased oxidative stress, mitochondrial dysfunction, cardiac remodeling, and exaggeration of HF [31,32]. Despite the detrimental effects of the accumulated BCAA and BCKA on the cardiac function observed in the mouse models, BCAA supplementation in rats and humans with HF proved beneficial, manifested by an improvement in exercise capacity and the patients’ overall fitness, as well as an elevation of albumin serum levels in HF patients with hypoalbuminemia [33,34]. The beneficial effects of BCAA in HF were further demonstrated by a positive correlation of the left ventricular ejection fraction with a ratio of BCAA to aromatic amino acids in systolic heart failure [35], as well as with a ratio of BCAA and total amino acids in patients with non-ischemic dilated cardiomyopathy [36]. 

Considering the beneficial and protective effects of BCAA supplementation, it is conceivable that valine actively counteracts the underlying AHF pathophysiology. More specifically, higher valine serum levels may ensure a better supply of valine to the failing heart, skeletal muscle, and other organs. By serving as a substrate for the synthesis of cellular building blocks and energy molecules or by other, presently unknown, beneficial effects, valine decreases the susceptibility of tissues and organs to the detrimental effects of the AHF pathophysiology, thus facilitating the patients’ fitness and survival. Additionally, or alternatively, by capturing information on the degree of hemodynamic, liver, and kidney function impairment, as well as on the efficacy of intestinal nutrient absorption, nutritional state, and the extent of muscle wasting, valine depicts various aspects of the underlying AHF pathophysiology and thus independently predicts the outcome of AHF patients.

### Strengths and Limitations

A well-defined patient population, as well as careful and uniform collection, storage, and analyses of the serum samples using a robust and highly reproducible method (NMR spectroscopy), represent the major strengths of the present study. There are, however, several limitations: The study design only permits the examination of correlations but cannot establish causality for the relationship between valine and other parameters. Accordingly, the mechanistic relationship between valine and the underlying AHF pathophysiology could not be examined. Additionally, since we determined the metabolite levels, including valine, only at the time of hospital admission, our results represent only a snapshot of the patients’ metabolic constellations. Furthermore, since the patients’ nutritional state at admission is unknown, the impact of fasting/feeding on valine and other metabolites could not be assessed. The fact that the enrolled patients came from a single European country makes the generalizability of the present data uncertain. Considering the rather moderate number of available samples (N = 315), our results will need to be confirmed in larger AHF cohorts. 

## 5. Conclusions

Based on our results, we conclude that low baseline valine serum levels are associated with increased 1-year mortality in AHF patients and might thus be of prognostic value in AHF.

## Figures and Tables

**Table 1:** Baseline characteristics and laboratory data of AHF patients upon hospital admission.

	Alive (N = 197)	Deceased (N = 118)	All (N = 315)	*p*-Value
Demographics				
Age (years)	72.5 (10.4)	77.0 (10.1)	74.2 (10.5)	**<0.001**
Sex, Female	85 (43.1%)	51 (43.2%)	136 (43.2%)	1.000
Comorbidities				
Hypertension	186 (94.4%)	108 (91.5%)	294 (93.3%)	0.355
T2DM	76 (38.6%)	56 (47.5%)	132 (41.9%)	0.127
CAD	100 (50.8%)	56 (47.5%)	156 (49.5%)	0.642
CMP	173 (87.8%)	115 (97.5%)	288 (91.4%)	**0.003**
AF	98 (49.7%)	72 (61.0%)	170 (54.0%)	0.062
CKD	72 (36.5%)	71 (60.2%)	143 (45.4%)	**<0.001**
MetS	130 (66.0%)	87 (73.7%)	217 (68.9%)	0.168
Physical measures at admission				
MAP (mmHg)	108.1 (24.2)	96.0 (19.5)	103.6 (23.3)	**<0.001**
Heart rate (beats/min)	103.8 (25.8)	95.3 (27.5)	100.6 (26.7)	**0.006**
Respiratory rate (breaths/min)	29.3 (6.9)	28.8 (6.0)	29.1 (6.5)	0.474
BMI (kg/m^2^)	27.4 (24.9, 30.7)	29.1 (25.3, 32.8)	28.0 (25.0, 31.6)	0.067
Signs and symptoms				
Symptom duration (days)	5.0 (3.0–5.0)	5.0 (4.0–5.0)	5.0 (4.0–5.0)	**0.022**
Rales or crackles	193 (98.0%)	118 (100.0%)	311 (98.7%)	0.301
JVD	97 (49.2%)	77 (65.3%)	174 (55.2%)	**0.007**
Enlarged liver	95 (48.2%)	81 (68.6%)	176 (55.9%)	**<0.001**
Ascites	20 (10.2%)	29 (24.6%)	49 (15.6%)	**0.001**
Peripheral edema	114 (57.9%)	90 (76.3%)	204 (64.8%)	**<0.001**
NYHA class				0.305
3	13 (6.6%)	4 (3.4%)	17 (5.4%)	
4	184 (93.4%)	114 (96.6%)	298 (94.6%)	
AHF type				**0.003**
New onset AHF	24 (12.2%)	3 (2.5)%	27 (8.6%)	
AHF following CHF	173 (87.8%)	115 (97.5%)	288 (91.4%)	
Echocardiography				
LVEDd/BSA (mm/m^2^)	29.1 (4.9)	28.5 (5.2)	28.8 (5.0)	0.346
LVEF (%)	40.1 (11.9)	39.1 (12.6)	39.8 (12.1)	0.455
SPAP (mmHg)	47.0 (42.0–55.0)	50.0 (45.0–60.0)	50.0 (45.0–60.0)	**0.005**
AHF class				0.575
HFrEF, EF < 40%	88 (44.9%)	55 (51.4%)	143 (47.2%)	
HFmrEF, EF 41–49%	55 (28.1%)	26 (24.3%)	81 (26.7%)	
HFpEF, EF ≥ 50%	53 (27.3%)	26 (24.3%)	79 (26.1%)	
Laboratory test results at admission				
TC (mmol/L)	3.8 (3.1, 4.9)	3.3 (2.7, 4.1)	3.5 (2.9, 4.5)	**<0.001**
HDL-C (mmol/L)	1.1 (0.9, 1.4)	1.1 (0.8, 1.3)	1.1 (0.9, 1.3)	**0.022**
LDL-C (mmol/L)	2.0 (1.5–2.8)	1.7 (1.3, 2.4)	1.9 (1.4, 2.7)	**<0.001**
Triglycerides (mmol/L)	1.0 (0.8, 1.4)	1.0 (0.8, 1.2)	1.0 (0.8, 1.3)	0.099
Albumin (g/L)	38.2 (35.5, 42.0)	36.7 (33.8, 39.7)	37.8 (34.8, 41.3)	**0.009**
Total proteins (g/L)	67.0 (62.0, 72.0)	65.5 (61.0, 70.0)	67.0 (61.0, 72.0)	0.214
Bilirubin (µmol/L)	17.4 (11.0, 28.5)	17.2 (11.9, 29.2)	17.3 (11.1, 28.7)	0.336
AST (U/L)	28.0 (22.0, 42.0)	27.0 (18.2, 52.5)	28.0 (20.0, 44.5)	0.542
ALT (U/L)	25.0 (16.0, 41.0)	21.0 (14.0, 46.5)	25.0 (15.0, 42.0)	0.226
Glucose (mmol/L)	7.7 (6.0, 10.8)	8.1 (6.3, 11.6)	7.9 (6.1, 11.2)	0.267
Sodium (mmol/L)	140.0 (138.0, 142.0)	138.0 (135.0, 141.0)	140.0 (136.5, 142.0)	**<0.001**
Potassium (mmol/L)	4.5 (4.1, 4.8)	4.5 (4.1, 5.0)	4.5 (4.1, 4.8)	0.194
Chloride (mmol/L)	104.0 (101.0, 107.0)	100.0 (97.0, 104.0)	103.0 (99.0, 106.0)	**<0.001**
BUN (mmol/L)	8.3 (6.3, 12.3)	12.3 (8.9, 16.8)	9.6 (6.9, 14.4)	**<0.001**
Creatinine (µmol/L)	107.0 (86.0, 144.0)	131.5 (107.0, 164.0)	117.0 (90.5, 152.5)	**<0.001**
eGFR (ml/min/1.73 m^2^)	54.0 (36.1, 70.5)	38.4 (29.1, 52.1)	46.6 (32.3, 65.0)	**<0.001**
CK (U/L)	105.0 (65.0, 174.0)	78.0 (50.2, 147.5)	93.0 (58.0, 165.5)	**0.007**
LDH (U/L)	252.0 (217.0, 316.0)	283.0 (230.8, 372.2)	265.0 (218.5, 332.0)	**0.029**
hsTnI (ng/L)	39.0 (17.5, 136.5)	61.0 (30.0, 149.0)	46.0 (20.0, 143.2)	**0.039**
NT-proBNP (pg/mL)	5350.0 (3151.0, 10,691.0)	10,733.0 (5486.5, 18,385.5)	6692.0 (3531.0, 14,395.5)	**<0.001**
CRP (mg/L)	10.3 (4.9, 21.9)	24.9 (6.4, 47.3)	12.2 (5.5, 33.1)	**<0.001**
IL-6 (pg/mL)	22.1 (11.3, 44.8)	40.6 (17.1, 79.6)	25.1 (12.9, 60.1)	**<0.001**
Fibrinogen (g/L)	4.0 (3.4, 4.7)	4.0 (3.1, 4.9)	4.0 (3.4, 4.8)	0.469
Erythrocytes (×10^12^/L)	4.7 (4.4, 5.1)	4.4 (3.8, 4.9)	4.6 (4.2, 5.1)	**<0.001**
Hemoglobin (g/L)	138.0 (124.0, 150.0)	126.0 (111.0, 141.0)	134.0 (119.0, 148.0)	**<0.001**
pH	7.4 (7.3, 7.5)	7.4 (7.3, 7.4)	7.4 (7.3, 7.5)	0.709
pO_2_ (kPa)	8.8 (7.2, 10.4)	8.8 (7.3, 10.4)	8.8 (7.2, 10.4)	0.803
pCO_2_ (kPa)	5.2 (4.4, 6.3)	5.2 (4.5, 7.1)	5.2 (4.5, 6.4)	0.386
HCO_3_ (mmol/L)	23.9 (21.2, 27.0)	24.4 (21.3, 28.9)	23.9 (21.3, 27.4)	0.368

Data are presented as *n* (%), mean and standard deviation, or median and interquartile range (q1, q3). Differences between AHF patients who survived and those who died within 1 year after study inclusion were tested with the Fisher’s exact test, *t* test, or Mann–Whitney U test. *p*-values < 0.05 are considered significant and are depicted in bold. AF, atrial fibrillation; AHF, acute heart failure; ALT, alanine aminotransferase; AST, aspartate aminotransferase; BMI, body mass index; BUN, blood urea nitrogen; CAD, coronary artery disease; CHF, chronic heart failure; CK, creatine kinase; CKD, chronic kidney disease; CMP, cardiomyopathy; CRP, C-reactive protein; EF, ejection fraction; eGFR, estimated glomerular filtration rate; HDL-C, high-density lipoprotein cholesterol; HFrEF, heart failure with reduced ejection fraction; HFmrEF, heart failure with mildly reduced ejection fraction; HFpEF, heart failure with preserved ejection fraction; hsTnI, high-sensitivity troponin I; HDL-C, high-density lipoprotein cholesterol; JVD, jugular vein distension; LDH, lactate dehydrogenase; LDL-C, low-density lipoprotein cholesterol; LVEDd, left ventricle end-diastolic diameter; LVEF, left ventricular ejection fraction; MAP, mean arterial pressure; MetS, metabolic syndrome; NT-proBNP, N-terminal pro-brain natriuretic peptide; NYHA, New York Heart Association Functional Classification; pO_2_, partial oxygen pressure; pCO2, partial carbon dioxide pressure; SPAP, systolic pulmonary artery pressure; TC, total cholesterol; T2DM: diabetes mellitus type 2.

**Table 2:** Serum levels of valine in various groups of AHF patients.

		Valine (µmol/L)	*p*-Value
T2D	No (N = 183)	275.4 (225.8, 312.1)	**0.045**
	Yes (N = 132)	292.1 (249.6, 322.7)	
CAD	No (N = 159)	267.3 (218.0, 302.6)	**<0.001**
	Yes (N = 156)	293.3 (254.4, 332.1)	
CKD	No (N = 172)	295.0 (255.5, 331.5)	**<0.001**
	Yes (N = 143)	257.4 (212.3, 295.9)	
MetS	No (N = 98)	284.7 (229.0, 329.0)	0.616
	Yes (N = 217)	277.8 (242.4, 313.9)	
AF	No (N = 145)	287.8 (251.5, 322.7)	**0.033**
	Yes (N = 170)	276.6 (224.8, 314.5)	
Sign(s) *	No (N = 66)	303.0 (278.0, 340.5)	**<0.001**
	Yes (N = 249)	270.0 (228.8, 308.1)	
AHF type	New onset AHF (N = 27)	292.6 (258.1, 316.4)	0.288
	AHF following CHF (N = 288)	278.8 (234.1, 318.5)	

Data are presented as median and interquartile range (q1, q3). Differences in metabolite levels between the groups were tested with the Mann-Whitney U test. *p*-values < 0.05 were considered significant and are depicted in bold. AF, atrial fibrillation; AHF, acute heart failure; CAD, coronary artery disease; CHF, chronic heart failure; CKD, chronic kidney disease; MetS, metabolic syndrome; T2D, type 2 diabetes mellitus. * Any of the following: peripheral edema, enlarged liver, ascites, or jugular venous distension.

## Data Availability

The data and analytic methods will be made available to other researchers on request.

## Supplementary Materials

The following supporting information can be downloaded at: https://www.mdpi.com/article/10.3390/biom13091323/s1, Scheme S1: Study flow chart; Table S1: Chronic medication of AHF patients prior to index hospitalization; Table S2: Integrals of NMR signals (arbitrary units) in patients who were alive compared to those who died within 1 year after hospitalization for AHF; Table S3: Serum levels (concentrations) of metabolites quantified by NMR spectroscopy that were significantly different between patients who were alive compared to those who died within 1 year after hospitalization fMEor AHF; Table S4: Univariable Cox regression analyses of parameters used for adjustment in the multivariable models; Table S5: Cox regression analyses of valine as a predictor of 1-year mortality in AHF patients; Figure S1: Receiver operating characteristics curve for prediction of death within 1 year of a panel of metabolites comprising valine, glutamic acid, ketoleucine, aspartic acid, trimethylamine N-oxide, creatine, phosphorylcholine, and tryptophan.

## References

[1] Bragazzi N.L., Zhong W., Shu J., Abu Much A., Lotan D., Grupper A., Younis A., Dai H. (2021). Burden of heart failure and underlying causes in 195 countries and territories from 1990 to 2017. Eur. J. Prev. Cardiol..

[2] Wende A.R., Brahma M.K., McGinnis G.R., Young M.E. (2017). Metabolic Origins of Heart Failure. JACC Basic. Transl. Sci..

[3] Pasini E., Aquilani R., Testa C., Baiardi P., Angioletti S., Boschi F., Verri M., Dioguardi F. (2016). Pathogenic Gut Flora in Patients with Chronic Heart Failure. JACC Heart Fail..

[4] Mamic P., Chaikijurajai T., Tang W.H.W. (2021). Gut microbiome–A potential mediator of pathogenesis in heart failure and its comorbidities: State-of-the-art review. J. Mol. Cell. Cardiol..

[5] Hunter W.G., Kelly J.P., McGarrah R.W., Kraus W.E., Shah S.H. (2016). Metabolic Dysfunction in Heart Failure: Diagnostic, Prognostic, and Pathophysiologic Insights from Metabolomic Profiling. Curr. Heart Fail. Rep..

[6] Ponikowski P., Voors A.A., Anker S.D., Bueno H., Cleland J.G.F., Coats A.J.S., Falk V., Gonzalez-Juanatey J.R., Harjola V.P., Jankowska E.A. (2016). 2016 ESC Guidelines for the Diagnosis and Treatment of Acute and Chronic Heart Failure. Rev. Esp. Cardiol. Engl. Ed..

[7] Loungani R.S., Teerlink J.R., Metra M., Allen L.A., Butler J., Carson P.E., Chen C.W., Cotter G., Davison B.A., Eapen Z.J. (2020). Cause of Death in Patients with Acute Heart Failure: Insights from RELAX-AHF-2. JACC Heart Fail..

[8] Klobucar I., Degoricija V., Potocnjak I., Trbusic M., Pregartner G., Berghold A., Fritz-Petrin E., Habisch H., Madl T., Frank S. (2022). HDL-apoA-II Is Strongly Associated with 1-Year Mortality in Acute Heart Failure Patients. Biomedicines.

[9] Degoricija V., Klobucar I., Potocnjak I., Dokoza Teresak S., Vidovic L., Pregartner G., Berghold A., Habisch H., Madl T., Frank S. (2022). Cholesterol Content of Very-Low-Density Lipoproteins Is Associated with 1-Year Mortality in Acute Heart Failure Patients. Biomolecules.

[10] World Medical A. (2013). World Medical Association Declaration of Helsinki: Ethical principles for medical research involving human subjects. JAMA.

[11] Reisinger A.C., Posch F., Hackl G., Marsche G., Sourij H., Bourgeois B., Eller K., Madl T., Eller P. (2021). Branched-Chain Amino Acids Can Predict Mortality in ICU Sepsis Patients. Nutrients.

[12] Kouzu H., Katano S., Yano T., Ohori K., Nagaoka R., Inoue T., Takamura Y., Ishigo T., Watanabe A., Koyama M. (2021). Plasma amino acid profiling improves predictive accuracy of adverse events in patients with heart failure. ESC Heart Fail..

[13] Conners K.M., Shearer J.J., Joo J., Park H., Manemann S.M., Remaley A.T., Otvos J.D., Connelly M.A., Sampson M., Bielinski S.J. (2023). The Metabolic Vulnerability Index: A Novel Marker for Mortality Prediction in Heart Failure. JACC Heart Fail..

[14] Hiraiwa H., Okumura T., Kondo T., Kato T., Kazama S., Ishihara T., Iwata E., Shimojo M., Kondo S., Aoki S. (2020). Usefulness of the plasma branched-chain amino acid/aromatic amino acid ratio for predicting future cardiac events in patients with heart failure. J. Cardiol..

[15] Hiraiwa H., Okumura T., Kondo T., Kato T., Kazama S., Kimura Y., Ishihara T., Iwata E., Shimojo M., Kondo S. (2021). Prognostic value of leucine/phenylalanine ratio as an amino acid profile of heart failure. Heart Vessel..

[16] Ambrosy A.P., Pang P.S., Khan S., Konstam M.A., Fonarow G.C., Traver B., Maggioni A.P., Cook T., Swedberg K., Burnett J.C. (2013). Clinical course and predictive value of congestion during hospitalization in patients admitted for worsening signs and symptoms of heart failure with reduced ejection fraction: Findings from the EVEREST trial. Eur. Heart J..

[17] Voors A.A., Ouwerkerk W., Zannad F., van Veldhuisen D.J., Samani N.J., Ponikowski P., Ng L.L., Metra M., Ter Maaten J.M., Lang C.C. (2017). Development and validation of multivariable models to predict mortality and hospitalization in patients with heart failure. Eur. J. Heart Fail..

[18] Lin Y., Pang L., Huang S., Shen J., Wu W., Tang F., Su W., Zhu X., Sun J., Quan R. (2022). The prevalence and survival of pulmonary hypertension due to left heart failure: A retrospective analysis of a multicenter prospective cohort study. Front. Cardiovasc. Med..

[19] Kalantar-Zadeh K., Block G., Horwich T., Fonarow G.C. (2004). Reverse epidemiology of conventional cardiovascular risk factors in patients with chronic heart failure. J. Am. Coll. Cardiol..

[20] Aquilani R., La Rovere M.T., Febo O., Baiardi P., Boschi F., Iadarola P., Viglio S., Dossena M., Bongiorno A.I., Pastoris O. (2012). Lung anabolic activity in patients with chronic heart failure: Potential implications for clinical practice. Nutrition.

[21] Flahault A., Metzger M., Chasse J.F., Haymann J.P., Boffa J.J., Flamant M., Vrtovsnik F., Houillier P., Stengel B., Thervet E. (2016). Low Serum Creatine Kinase Level Predicts Mortality in Patients with a Chronic Kidney Disease. PLoS ONE.

[22] Pasini E., Aquilani R., Dioguardi F.S., D’Antona G., Gheorghiade M., Taegtmeyer H. (2008). Hypercatabolic syndrome: Molecular basis and effects of nutritional supplements with amino acids. Am. J. Cardiol..

[23] Holecek M. (2018). Branched-chain amino acids in health and disease: Metabolism, alterations in blood plasma, and as supplements. Nutr. Metab..

[24] Hiraiwa H., Okumura T., Murohara T. (2023). Amino acid profiling to predict prognosis in patients with heart failure: An expert review. ESC Heart Fail..

[25] Holecek M., Sprongl L., Skopec F., Andrys C., Pecka M. (1997). Leucine metabolism in TNF-alpha- and endotoxin-treated rats: Contribution of hepatic tissue. Am. J. Physiol..

[26] Hasper D., Hummel M., Kleber F.X., Reindl I., Volk H.D. (1998). Systemic inflammation in patients with heart failure. Eur. Heart J..

[27] She P., Van Horn C., Reid T., Hutson S.M., Cooney R.N., Lynch C.J. (2007). Obesity-related elevations in plasma leucine are associated with alterations in enzymes involved in branched-chain amino acid metabolism. Am. J. Physiol. Endocrinol. Metab..

[28] Kuzuya T., Katano Y., Nakano I., Hirooka Y., Itoh A., Ishigami M., Hayashi K., Honda T., Goto H., Fujita Y. (2008). Regulation of branched-chain amino acid catabolism in rat models for spontaneous type 2 diabetes mellitus. Biochem. Biophys. Res. Commun..

[29] Garibotto G., Paoletti E., Fiorini F., Russo R., Robaudo C., Deferrari G., Tizianello A. (1993). Peripheral metabolism of branched-chain keto acids in patients with chronic renal failure. Miner. Electrolyte Metab..

[30] Shah S.H., Bain J.R., Muehlbauer M.J., Stevens R.D., Crosslin D.R., Haynes C., Dungan J., Newby L.K., Hauser E.R., Ginsburg G.S. (2010). Association of a peripheral blood metabolic profile with coronary artery disease and risk of subsequent cardiovascular events. Circ. Cardiovasc. Genet..

[31] Sun H., Olson K.C., Gao C., Prosdocimo D.A., Zhou M., Wang Z., Jeyaraj D., Youn J.Y., Ren S., Liu Y. (2016). Catabolic Defect of Branched-Chain Amino Acids Promotes Heart Failure. Circulation.

[32] Wang W., Zhang F., Xia Y., Zhao S., Yan W., Wang H., Lee Y., Li C., Zhang L., Lian K. (2016). Defective branched chain amino acid catabolism contributes to cardiac dysfunction and remodeling following myocardial infarction. Am. J. Physiol. Heart Circ. Physiol..

[33] Tanada Y., Shioi T., Kato T., Kawamoto A., Okuda J., Kimura T. (2015). Branched-chain amino acids ameliorate heart failure with cardiac cachexia in rats. Life Sci..

[34] Uchino Y., Watanabe M., Takata M., Amiya E., Tsushima K., Adachi T., Hiroi Y., Funazaki T., Komuro I. (2018). Effect of Oral Branched-Chain Amino Acids on Serum Albumin Concentration in Heart Failure Patients with Hypoalbuminemia: Results of a Preliminary Study. Am. J. Cardiovasc. Drugs.

[35] Hakuno D., Hamba Y., Toya T., Adachi T. (2015). Plasma amino acid profiling identifies specific amino acid associations with cardiovascular function in patients with systolic heart failure. PLoS ONE.

[36] Kimura Y., Okumura T., Kazama S., Shibata N., Oishi H., Arao Y., Kuwayama T., Kato H., Yamaguchi S., Hiraiwa H. (2020). Usefulness of Plasma Branched-Chain Amino Acid Analysis in Predicting Outcomes of Patients with Nonischemic Dilated Cardiomyopathy. Int. Heart J..

