# Supplementary material for: Low Valine Serum Levels Predict Increased 1-Year Mortality in Acute Heart Failure Patients
Source: Biomolecules. 2023 Aug 29;13(9):1323. doi: 10.3390/biom13091323 (PMC10527293; doi:10.3390/biom13091323)
Supplement: Supplementary file 1 [file biomolecules-13-01323-s001.zip › Figure S1.pdf]

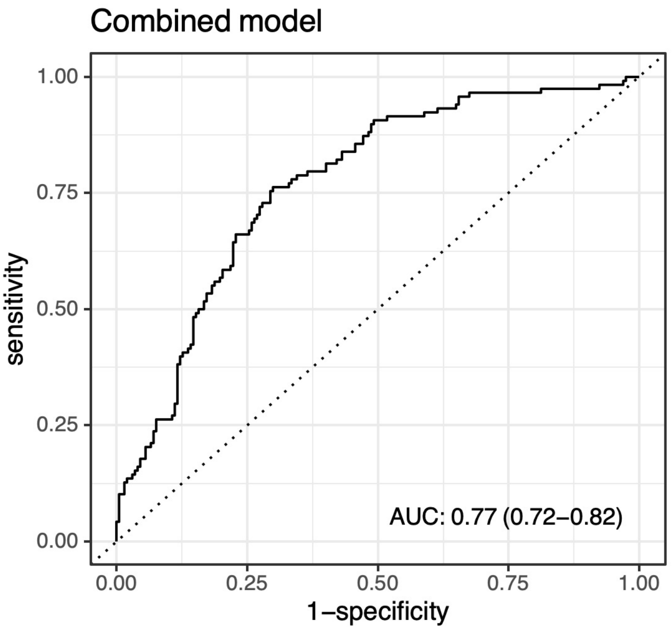

Figure S1. Receiver operating characteristics curve for prediction of death within 1 year of a panel of metabolites comprising valine, glutamic acid, ketoleucine, aspartic acid, trimethylamine N-oxide, creatine, phosphorylcholine, and tryptophan.
