# Supplementary material for: Low Valine Serum Levels Predict Increased 1-Year Mortality in Acute Heart Failure Patients
Source: Biomolecules. 2023 Aug 29;13(9):1323. doi: 10.3390/biom13091323 (PMC10527293; doi:10.3390/biom13091323)
Supplement: Supplementary file 1 [file biomolecules-13-01323-s001.zip › Scheme S1.pdf]

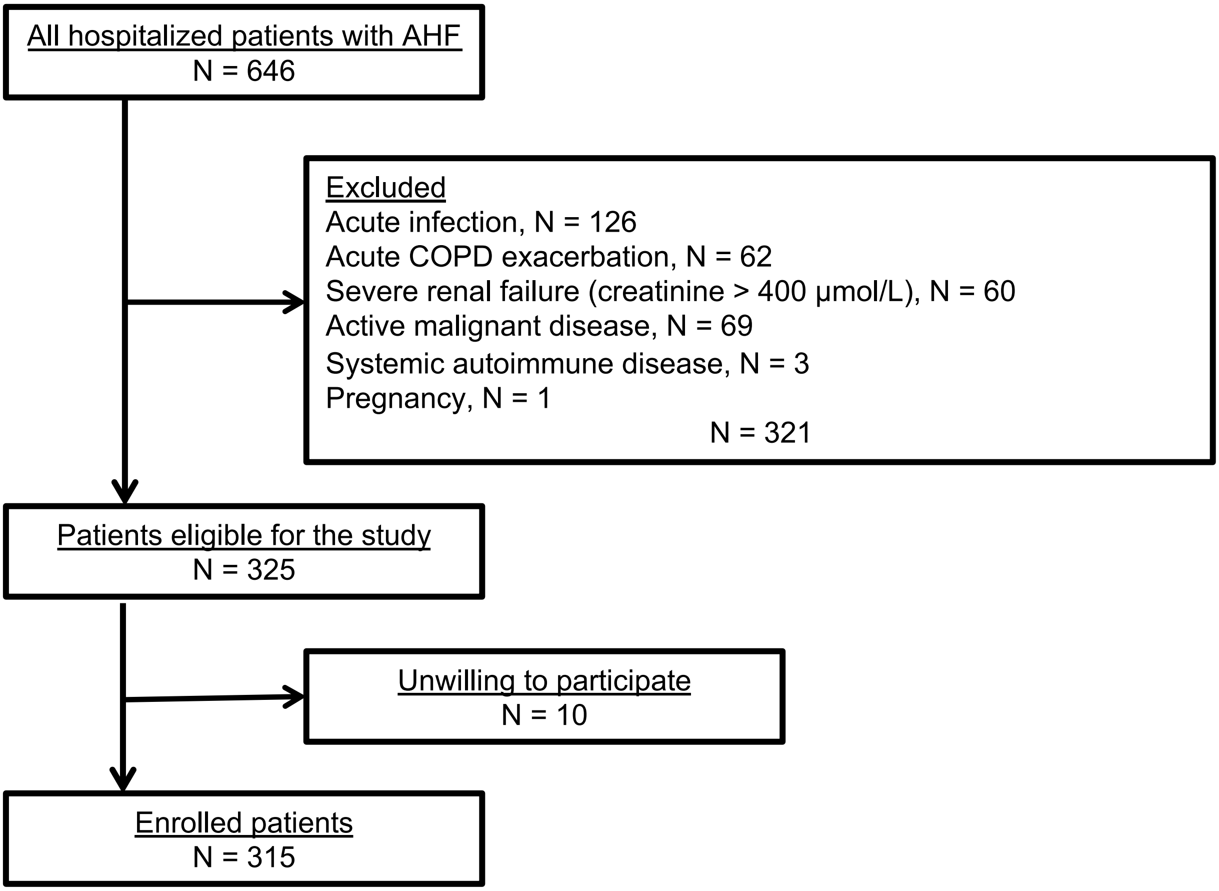
All hospitalized patients with AHF
N = 646
Excluded
Acute infection, N = 126
Acute COPD exacerbation, N = 62
Severe renal failure (creatinine > 400 µmol/L), N = 60
Active malignant disease, N = 69
Systemic autoimmune disease, N = 3
Pregnancy, N = 1
N = 321
Patients eligible for the study
N = 325
Unwilling to participate
N = 10
Enrolled patients
N = 315
