# Supplementary material for: Low Valine Serum Levels Predict Increased 1-Year Mortality in Acute Heart Failure Patients
Source: Biomolecules. 2023 Aug 29;13(9):1323. doi: 10.3390/biom13091323 (PMC10527293; doi:10.3390/biom13091323)
Supplement: Supplementary file 1 [file biomolecules-13-01323-s001.zip › Table S2.pdf]

Table S2. Integrals of NMR signals (arbitrary units) in patients who were alive compared to those who died within 1 year after hospitalization for AHF.

Metabolite (AU)	Alive (N=197)	Deceased (N=118)	All (N=315)	p-value
2-Hydroxy-3-methylbutyric acid	5.5 (4.7, 6.7)	5.7 (4.6, 6.8)	5.6 (4.7, 6.7)	0.792
Leucine	21.2 (18.7, 24.2)	20.5 (17.5, 23.7)	21.1 (18.1, 24.1)	0.138
Isoleucine	8.7 (7.3, 10.3)	7.9 (6.5, 9.5)	8.4 (7.1, 9.8)	0.008
Valine	27.6 (23.9, 30.8)	24.3 (19.8, 27.9)	26.4 (22.2, 30.1)	**< 0.001**
Isobutyric acid	3.3 (2.7, 3.9)	3.2 (2.7, 4.1)	3.3 (2.7, 4.0)	0.956
3-Methy2-oxovaleric acid	138.8 (95.8, 206.6)	150.3 (108.8, 221.9)	143.8 (101.3, 217.8)	0.394
Ethanol	4.2 (3.9, 4.6)	4.2 (3.8, 4.7)	4.2 (3.9, 4.6)	0.892
3-Hydroxybutyric acid	30.8 (13.4, 58.7)	35.1 (18.4, 64.5)	32.2 (15.1, 62.5)	0.244
Lactic acid	358.2 (290.9, 467.5)	350.1 (277.0, 440.8)	354.3 (287.2, 460.0)	0.400
Alanine	42.9 (35.7, 52.2)	38.8 (30.9, 47.7)	41.2 (34.0, 49.9)	0.001
Sebacic acid	3.9 (3.5, 4.2)	3.9 (3.5, 4.4)	3.9 (3.5, 4.2)	0.126
Arginine	5.9 (5.4, 6.4)	5.7 (5.2, 6.2)	5.8 (5.4, 6.3)	0.035
Acetic acid	23.6 (15.6, 45.7)	19.0 (15.2, 38.9)	22.1 (15.5, 43.3)	0.077
Acetone	6.7 (5.4, 10.5)	7.2 (5.6, 10.6)	6.9 (5.5, 10.5)	0.188
Glutamic acid	26.8 (24.7, 29.9)	24.6 (22.5, 26.4)	25.9 (24.0, 28.8)	**< 0.001**
Succinic acid	9.5 (7.6, 11.4)	9.5 (7.7, 11.5)	9.5 (7.6, 11.5)	0.995
Glutamine	43.6 (39.0, 48.9)	42.1 (36.7, 50.6)	43.1 (38.3, 49.5)	0.433
Citric acid	7.5 (6.2, 9.9)	7.4 (6.2, 9.5)	7.4 (6.2, 9.7)	0.925
Ketoleucine	2.5 (2.2, 3.1)	2.3 (1.9, 2.7)	2.4 (2.1, 3.0)	**< 0.001**
Methionine	1.5 (1.4, 1.8)	1.6 (1.4, 1.9)	1.6 (1.4, 1.8)	0.033
Aspartic acid	1.9 (1.7, 2.2)	2.1 (1.9, 2.4)	2.0 (1.8, 2.3)	**< 0.001**
Dimethylamine	2.1 (1.7, 4.2)	2.5 (1.9, 4.5)	2.2 (1.8, 4.3)	0.023
Trimethylamine	2.2 (2.0, 2.4)	2.3 (2.1, 2.5)	2.3 (2.1, 2.4)	0.012
Lysine	7.4 (6.6, 8.2)	7.3 (6.7, 8.4)	7.4 (6.6, 8.3)	0.397
Creatine	26.9 (23.4, 31.6)	30.0 (26.6, 36.9)	28.4 (24.8, 33.8)	**< 0.001**
Ornithine	7.3 (6.5, 8.6)	7.5 (6.3, 8.6)	7.4 (6.3, 8.6)	0.894
Choline	14.9 (12.2, 16.8)	15.0 (12.6, 17.4)	14.9 (12.3, 17.0)	0.287
Phosphorylcholine	22.7 (18.6, 27.5)	18.7 (16.1, 21.9)	21.2 (17.3, 25.8)	**< 0.001**
Glycerophosphocholine	61.9 (53.6, 71.5)	59.9 (51.8, 69.3)	61.1 (53.0, 71.2)	0.390
Carnitine	54.9 (45.3, 63.6)	54.9 (44.9, 61.8)	54.9 (45.2, 62.8)	0.768
Trimethylamine N-oxide	9.8 (8.7, 11.6)	9.1 (7.8, 10.2)	9.5 (8.4, 11.0)	**< 0.001**
Glycine	31.5 (27.6, 37.8)	32.0 (26.9, 36.5)	31.5 (27.4, 37.1)	0.831
Glycerol	28.1 (25.3, 31.9)	28.9 (24.7, 32.4)	28.1 (25.2, 31.9)	0.729
myo-Inositol	10.1 (8.7, 12.6)	10.9 (9.4, 14.1)	10.5 (8.9, 13.2)	0.005
Betaine	14.8 (13.1, 17.1)	14.6 (12.6, 17.4)	14.7 (12.9, 17.2)	0.520
Serine	11.7 (10.3, 13.3)	11.5 (10.2, 13.0)	11.5 (10.3, 13.2)	0.498
Threonine	2.5 (2.1, 2.7)	2.3 (2.1, 2.7)	2.4 (2.1, 2.7)	0.233
Mannose	4.7 (4.0, 5.7)	5.0 (4.1, 6.3)	4.9 (4.0, 6.0)	0.100
Glucose	190.3 (159.5, 248.0)	189.9 (151.5, 252.7)	189.9 (157.5, 248.3)	0.624

Fumaric acid*	0.2 (0.1, 0.3)	0.2 (0.1, 0.2)	0.2 (0.1, 0.3)	0.234
4-Aminohippuric acid**	0.4 (0.3, 0.5)	0.4 (0.3, 0.6)	0.4 (0.3, 0.5)	0.002
Tyrosine	4.0 (3.4, 4.8)	4.1 (3.3, 4.7)	4.0 (3.4, 4.8)	0.935
Histidine***	0.4 (0.3, 0.6)	0.5 (0.3, 0.8)	0.4 (0.3, 0.7)	0.028
Phenylalanine	5.2 (4.6, 5.8)	5.3 (4.6, 5.9)	5.2 (4.6, 5.9)	0.537
Indoxyl sulfate****	0.2 (0.1, 0.3)	0.3 (0.2, 0.3)	0.2 (0.1, 0.3)	0.025
Tryptophan	1.9 (1.6, 2.3)	1.7 (1.4, 2.0)	1.8 (1.5, 2.2)	**< 0.001**
Salicylic acid*****	0.4 (0.2, 0.8)	0.4 (0.2, 0.9)	0.4 (0.2, 0.8)	0.817
Hippuric acid******	0.5 (0.4, 0.8)	0.6 (0.3, 0.9)	0.5 (0.3, 0.8)	0.743
Formic acid	1.1 (0.7, 3.1)	1.5 (0.8, 2.6)	1.3 (0.8, 3.0)	0.508

Data are presented as median and interquartile range (q1, q3). Differences between AHF patients who were alive and those who deceased within 1 year after hospitalization for AHF were tested using the Mann-Whitney U test.

Due to insufficient quality of the NMR signals *8 (4 alive, 4 deceased), **23 (20 alive, 3 deceased),***37 (27 alive, 10 deceased), ****38 (26 alive, 12 deceased), *****18 (15 alive, 3 deceased), and ******13 (8 alive, 5 deceased) samples were excluded from the analysis.

P-values of < 0.001 were considered significant after a Bonferroni correction for multiple testing and are depicted in bold.

AU, arbitrary units.
