# Supplementary material for: Low Valine Serum Levels Predict Increased 1-Year Mortality in Acute Heart Failure Patients
Source: Biomolecules. 2023 Aug 29;13(9):1323. doi: 10.3390/biom13091323 (PMC10527293; doi:10.3390/biom13091323)
Supplement: Supplementary file 1 [file biomolecules-13-01323-s001.zip › Table S3.pdf]

Table S3. Serum levels (concentrations) of metabolites quantified by NMR spectroscopy which were significantly different in patients who were alive compared to those who died within 1 year after hospitalization for AHF.

Metabolite (μmol/L)	Alive (N=197)	Deceased (N=118)	All (N=315)	p-value
Valine	292.2 (252.9, 325.7)	257.0 (209.5, 295.1)	279.2 (234.9, 318.8)	**< 0.001**
Glutamic acid	473.1 (436.3, 527.2)	433.2 (396.9, 466.6)	457.8 (422.8, 508.6)	**< 0.001**
Ketoleucine	39.7 (34.7, 48.9)	35.7 (30.0, 43.0)	38.4 (33.4, 47.3)	**< 0.001**
Aspartic acid	123.7 (109.9, 137.9)	133.7 (117.8, 150.3)	128.3 (113.4, 144.2)	**< 0.001**
Trimethylamine N-oxide	17.4 (15.3, 20.5)	16.0 (13.7, 17.9)	16.7 (14.8, 19.5)	**< 0.001**
Creatine	142.3 (123.8, 167.3)	158.6 (140.6, 195.2)	150.5 (131.4, 178.9)	**< 0.001**
Phosphorylcholine	40.1 (32.9, 48.6)	32.9 (28.4, 38.7)	37.3 (30.5, 45.4)	**< 0.001**
Tryptophan	60.6 (51.0, 72.8)	52.6 (43.7, 62.5)	57.8 (46.3, 70.1)	**< 0.001**

Data are presented as median and interquartile range (q1, q3). Differences between AHF patients who were alive and those who deceased within 1 year after hospitalization for AHF were tested using the Mann-Whitney U test.

P-values of < 0.001 were considered significant after a Bonferroni correction for multiple testing and are depicted in bold.
