# Supplementary material for: Low Valine Serum Levels Predict Increased 1-Year Mortality in Acute Heart Failure Patients
Source: Biomolecules. 2023 Aug 29;13(9):1323. doi: 10.3390/biom13091323 (PMC10527293; doi:10.3390/biom13091323)
Supplement: Supplementary file 1 [file biomolecules-13-01323-s001.zip › Table S4.pdf]

Table S4. Univariable Cox regression analyses of parameters used for adjustment in the multivariable models.

	HR (95% CI)	p-value	Events/N
Age (years)	1.04 (1.02-1.06)	**<0.001**	118/315
Female sex	0.99 (0.69-1.43)	0.973	118/315
BMI (kg/m^2)	1.03 (1.00-1.06)	**0.031**	118/315
MAP (mmHg)	0.98 (0.97-0.99)	**<0.001**	118/315
NT-proBNP (pg/mL)	1.00 (1.00-1.00)	**<0.001**	118/315
Haemoglobin (g/L)	0.98 (0.97-0.98)	**<0.001**	118/315
BUN (mmol/L)	1.07 (1.05-1.10)	**<0.001**	117/314
eGFR (mL/min/1.73m^2)	0.98 (0.97-0.99)	**<0.001**	118/315
CRP (mg/L)	1.01 (1.00-1.01)	**<0.001**	118/315
ALT (U/L)	1.00 (1.00-1.00)	**0.001**	118/315
Albumin (g/L)	0.96 (0.92-0.99)	**0.012**	113/304
Total cholesterol (mmol/L)	0.71 (0.60-0.84)	**<0.001**	118/315

P-values < 0.05 were considered significant and are depicted in bold.

AHF, acute heart failure; ALT, alanine aminotransferase; BMI, body mass index; BUN, blood urea nitrogen; CI, confidence interval; CRP, C-reactive protein; eGFR, estimated glomerular filtration rate; HR, hazard ratio; MAP, mean arterial pressure; N, number of observations; NT-proBNP, N-terminal pro-brain natriuretic peptide.
