# Supplementary material for: Low Valine Serum Levels Predict Increased 1-Year Mortality in Acute Heart Failure Patients
Source: Biomolecules. 2023 Aug 29;13(9):1323. doi: 10.3390/biom13091323 (PMC10527293; doi:10.3390/biom13091323)
Supplement: Supplementary file 1 [file biomolecules-13-01323-s001.zip › Table S5.pdf]

**Table S5.** Cox regression analyses of valine as predictor of 1-year mortality in AHF patients.

| | **HR (95% CI) per 1-SD** | **p-value** | **Event/N** |
|---|---|---|---|
| Base model | 0.73 (0.59-0.90) | **0.003** | 112/303 |
| Model 1 | 0.73 (0.59-0.89) | **0.002** | 112/303 |
| Model 2 | 0.73 (0.60-0.90) | **0.003** | 112/303 |
| Model 3 | 0.74 (0.60-0.91) | **0.004** | 112/303 |
| Model 4 | 0.73 (0.59-0.89) | **0.002** | 112/303 |
| Model 5 | 0.74 (0.61-0.90) | **0.003** | 117/314 |
| Model 6 | 0.75 (0.61-0.91) | **0.003** | 117/314 |

Valine ($\mu$mol/L) was log-transformed before Cox regression analysis.

Base model: Adjusted for age, sex, BMI, MAP, eGFR, BUN, CRP, NT-proBNP, hemoglobin, ALT, albumin, and total cholesterol.

Model 1: Base model with LDL-cholesterol instead of total cholesterol.

Model 2: Base model with signs* of venous volume overload instead of total cholesterol.

Model 3: Base model with previous ß-blocker use instead of total cholesterol.

Model 4: Base model with previous ACEI use instead of total cholesterol.

Model 5: Base model without albumin.

Model 6: Base model with signs* of venous volume overlaod instead of albumin.

*Signs include any of the following: peripheral edema, enlarged liver, ascites, or jugular venous distension.

P-values of < 0.05 were considered significant and are depicted in bold.

ACEI: angiotensin-converting-enzyme inhibitor; ALT, alanine aminotransferase; BMI, body mass index; BUN, blood urea nitrogen; CRP, C-reactive protein; eGFR, estimated glomerular filtration rate; HR, hazard ratio; MAP, mean arterial pressure; N, number of observations; NT-proBNP, N-terminal pro-brain natriuretic peptide.
